# Gene model for the ortholog of *Roc1a* in *Drosophila ananassae*

**DOI:** 10.17912/micropub.biology.001036

**Published:** 2025-06-04

**Authors:** Megan E. Lawson, Kelsey Gammage, Calvin Dexel, Lindsey J. Long, Chinmay P. Rele, Laura K. Reed

**Affiliations:** 1 The University of Alabama, Tuscaloosa, AL USA; 2 Oklahoma Christian University, Edmond, OK USA

## Abstract

Gene model for the ortholog of *Regulator of cullins 1a* ( *Roc1a* ) in the *Drosophila ananassae* May 2011 (Agencourt dana_caf1/DanaCAF1) Genome Assembly (GenBank Accession: GCA_000005115.1 ). This ortholog was characterized as part of a developing dataset to study the evolution of the Insulin/insulin-like growth factor signaling pathway (IIS) across the genus *Drosophila* using the Genomics Education Partnership gene annotation protocol for Course-based Undergraduate Research Experiences.

**Figure 1. Roc1a gene model comparison between Drosophila ananassae and Drosophila melanogaster orthologs:**
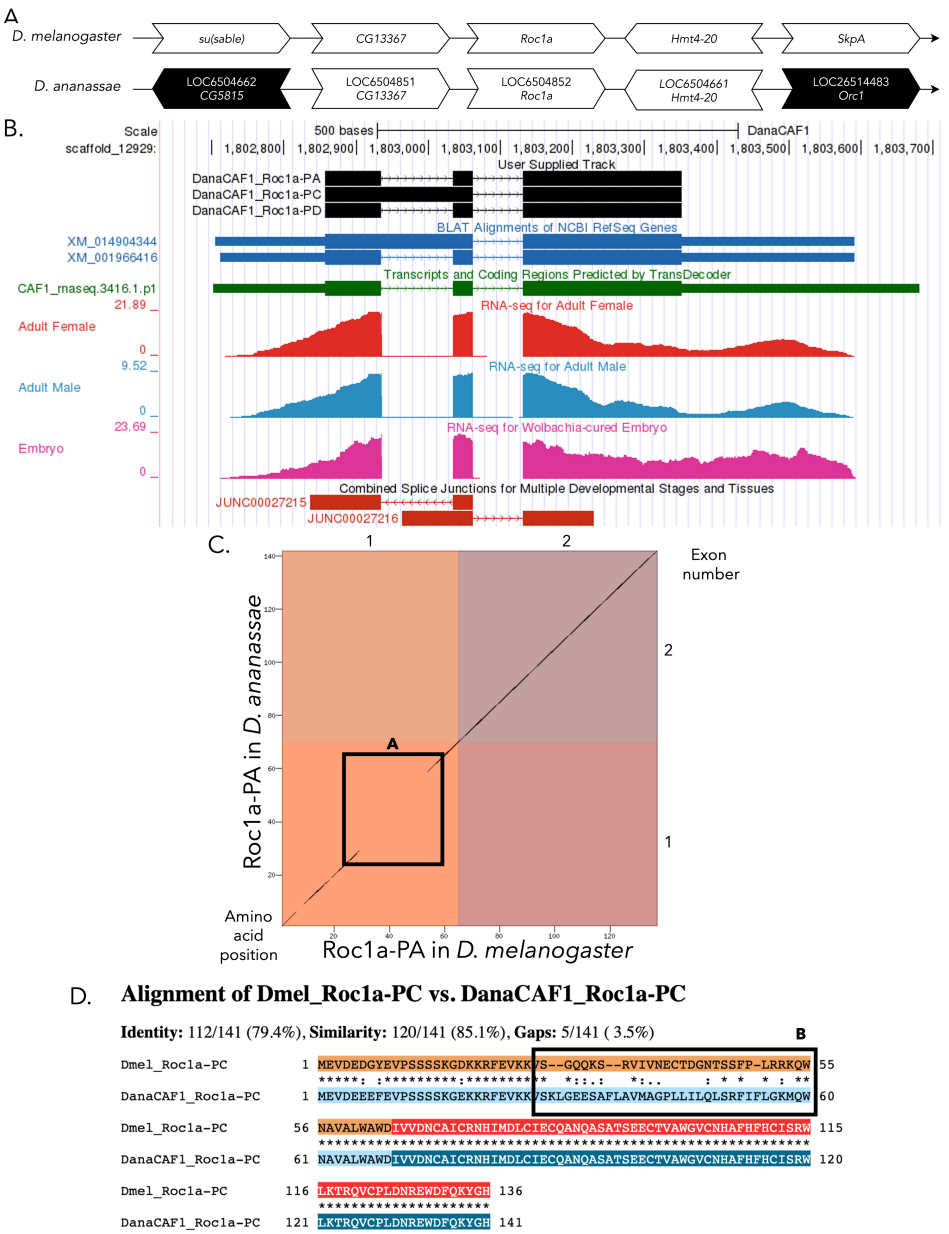
**
(A) Synteny comparison of the genomic neighborhoods for *Roc1a* in *Drosophila melanogaster* and *D.* *ananassae* .** Thin underlying arrows indicate the DNA strand within which the reference gene– *Roc1a* –is located in *D. melanogaster* (top) and *D. ananassae* (bottom). Thin arrows pointing to the right indicate that *Roc1a* is on the positive (+) strand in *D. melanogaster* and *D. ananassae* . The wide gene arrows pointing in the same direction as *Roc1a* are on the same strand relative to the thin underlying arrows, while wide gene arrows pointing in the opposite direction of *Roc1a* are on the opposite strand relative to the thin underlying arrows. White gene arrows in *D. ananassae* indicate orthology to the corresponding gene in *D. melanogaster* . Gene symbols given in the *D. ananassae* gene arrows indicate the orthologous gene in *D. melanogaster* , while the locus identifiers are specific to *D. ananassae* . **(B) Gene Model in GEP UCSC Track Data Hub** (Raney et al. 2014). The coding-regions of *Roc1a* in *D. ananassae* are displayed in the User Supplied Track (black); coding CDSs are depicted by thick rectangles and introns by thin lines with arrows indicating the direction of transcription. Subsequent evidence tracks include BLAT Alignments of NCBI RefSeq Genes (dark blue, alignment of Ref-Seq genes for *D. ananassae* ), Transcripts and Coding Regions Predicted by TransDecoder (dark green), RNA-Seq from Adult Females, Adult Males, and Wolbachia- cured embryos (red, blue, and pink, respectively; alignment of Illumina RNA-Seq reads from *D. ananassae* ), and Splice Junctions Predicted by regtools using *D. ananassae* RNA-Seq (Gravely et al. 2011; SRP006203 , PRJNA257286 , SRP007906 , PRJNA388952 ). Splice junctions shown have a minimum read-depth of 10 with >1000 supporting reads indicated in red. **(C) Dot Plot of Roc1a-PC in *D. melanogaster* ( *x* -axis) vs. the orthologous peptide in *D. ananassae* ( *y* -axis).** Amino acid number is indicated along the left and bottom; CDS number is indicated along the top and right, and CDSs are also highlighted with alternating colors. Box A highlights a gap in the alignment caused by low sequence similarity between this region of Roc1a-PC in *D. ananassae* compared to *D. melanogaster.* **(D) Protein alignment of Roc1a-PC in *D. melanogaster* (top row) vs. the orthologous peptide in *D. ananassae* (bottom row).** The amino acids in Roc1a-RC are indicated in each species' respective row, with CDSs being highlighted in alternating colors. Asterisks between the protein alignment from each species indicate that the amino acid at that position is the same, colons and periods indicate the amino acids are very similar structurally (colons at a higher extent and periods at a lesser extent), and empty space indicates the amino acids are not similar. Lines in the protein sequence in either species indicate that there are gaps in that portion of the alignment for that species. Box 1D-B indicates the same region of decreased sequence similarity in CDS one of *Roc1a-RC* that is highlighted in Box 1C-A.

## Description

**Table:** 

* This article reports a predicted gene model generated by undergraduate work using a structured gene model annotation protocol defined by the Genomics Education Partnership (GEP; thegep.org ) for Course-based Undergraduate Research Experience (CURE). The following information in this box may be repeated in other articles submitted by participants using the same GEP CURE protocol for annotating Drosophila species orthologs of Drosophila melanogaster genes in the insulin signaling pathway. * "In this GEP CURE protocol students use web-based tools to manually annotate genes in non-model *Drosophila* species based on orthology to genes in the well-annotated model organism fruitfly *Drosophila melanogaster* . The GEP uses web-based tools to allow undergraduates to participate in course-based research by generating manual annotations of genes in non-model species (Rele et al., 2023). Computational-based gene predictions in any organism are often improved by careful manual annotation and curation, allowing for more accurate analyses of gene and genome evolution (Mudge and Harrow 2016; Tello-Ruiz et al., 2019). These models of orthologous genes across species, such as the one presented here, then provide a reliable basis for further evolutionary genomic analyses when made available to the scientific community.” (Myers et al., 2024). “The particular gene ortholog described here was characterized as part of a developing dataset to study the evolution of the Insulin/insulin-like growth factor signaling pathway (IIS) across the genus *Drosophila* . The Insulin/insulin-like growth factor signaling pathway (IIS) is a highly conserved signaling pathway in animals and is central to mediating organismal responses to nutrients (Hietakangas and Cohen 2009; Grewal 2009).” (Myers et al., 2024). “ *Roc1a (* also known as *Rbx1* ) is a member of the SCF E3 ubiquitin ligase complex and was originally identified through sequence similarity with vertebrate and yeast homologs and biochemical interaction studies (Bocca et al., 2001). *Roc1a* deletion mutants are lethal between the first and second larval instars and *Roc1a* mutant clones in imaginal discs have cell proliferation defects (Noureddine et al., 2002). In addition, Roc1a and other members of the SCF E3 ubiquitin ligase complex function in the pruning of larval neurons by targeting the insulin-responsive kinase Akt for ubiquitination and degradation, thus inhibiting insulin signaling (Wong et al., 2013).” (Lawson et al., 2025). “ *D* . *ananassae* (NCBI:txid7217) is part of the *melanogaster* species group within the subgenus *Sophophora* of the genus *Drosophila* (Sturtevant 1939; Bock and Wheeler 1972). It was first described by Doeschall (1858). *D. ananassae* is circumtropical (Markow and O'Grady 2005; https://www.taxodros.uzh.ch, accessed 1 Feb 2023), and often associated with human settlement (Singh 2010). It has been extensively studied as a model for its cytogenetic and genetic characteristics, and in experimental evolution (Kikkawa 1938; Singh and Yadav 2015).” (Lawson et al., 2024).

We propose a gene model for the *D. ananassae* ortholog of the *D. melanogaster* *Regulator of cullins 1a* ( *Roc1a* ) gene. The genomic region of the ortholog corresponds to the uncharacterized protein LOC6504852 (RefSeq accession XP_014759830.1 ) in the May 2011 (Agencourt dana_caf1/DanaCAF1) Genome Assembly of *D. ananassae* (GenBank Accession: GCA_000005115.1 ; Drosophila 12 Genomes Consortium et al., 2007). This model is based on RNA-Seq data from *D. ananassae* (Gravely et al. 2011; SRP006203 , PRJNA257286 , SRP007906 , PRJNA388952 *)* and *Roc1a* in *D. melanogaster* using FlyBase release FB2023_02 ( GCA_000001215.4 ; Larkin et al. 2021; Gramates et al., 2022; Jenkins et al., 2022).

***Synteny***

The reference gene, *Roc1a ,* occurs on chromosome X in *D. melanogaster* and is flanked upstream by *CG13367* and *suppressor of sable* ( *Su(sable)* ) and downstream by *Histone methyltransferase 4-20* ( *Hmt4-20* ) and *SKP1-related A* *( SkpA )* . The *tblastn* search of *D. melanogaster* Roc1a-PC (query) against the *D. ananassae* (GenBank Accession: GCA_000005115.1 ) Genome Assembly (database) placed the putative ortholog of *Roc1a* within scaffold_12929 ( CH902632.1 ) at locus LOC6504852 ( XP_014759830.1 )— with an E-value of 2e-63 and a percent identity of 68.29%. Furthermore, the putative ortholog is flanked upstream by LOC6504851 ( XP_001966451.1 ) and LOC6504662 ( XP_001966450.2 ), which correspond to *CG13367* and *CG5815* in *D. melanogaster* (E-value: 8e-176 and 0.0; identity: 68.19% and 67.55%, respectively, as determined by *blastp* ; Figure 1A, Altschul et al., 1990). The putative ortholog of *Roc1a* is flanked downstream by LOC6504661 ( XP_032309073.1 ) and LOC26514483 ( XP_014759887.1 ), which correspond to *Hmt4-20* and *Ocrl* in *D. melanogaster* (E-value: 0.0 and 0.0; identity: 67.40% and 69.68%, respectively, as determined by *blastp* ). The putative ortholog assignment for *Roc1a* in *D. ananassae* is supported by the partial conservation of the synteny of this genomic neighborhood, and all *BLAST* results indicate good-quality matches.

***Protein Model***

*Roc1a* in *D. ananassae* has two unique protein-coding isoforms: Roc1a-PA (identical to Roc1a-PD) and Roc1a-PC ( Figure 1B ). mRNA isoforms *Roc1a-RA* and *Roc1a-RD* contain three protein-coding CDSs. mRNA isoform *Roc1a-RC* has two CDSs, with its first CDS (FlyBase ID: 1_2094_0) spanning the length of the first two CDSs of *Roc1a-RA* and *Roc1a-RD* (FlyBase IDs: 2_2094_0 and 3_2094_0) combined (all CDS IDs based on FlyBase release FB2023_02; GCA_000001215.4 ). Relative to the ortholog in *D. melanogaster* , the RNA CDS number and isoform structure is conserved *.* The sequence of Roc1a-PC in *D. ananassae* has 79.43% identity (E-value: 2e-76) with the protein-coding isoform Roc1a-PC in *D. melanogaster* , as determined by *blastp* ( Figure 1C ). Coordinates of this curated gene model (Roc1a-PC, Roc1a-PA, Roc1a-PD) are stored by NCBI at GenBank/BankIt (accession **BK064627 , BK064628 , BK064629** , respectively). These data are also archived in the CaltechDATA repository (see “Extended Data” section below).

***Special characteristics of the protein model***

**Low sequence similarity in the latter portion of the first CDS of *Roc1a-RC***

Boxes 1C-A and 1D-B highlight a region of decreased sequence similarity in the alignment of the first CDS of *Roc1a-RC* in *D. melanogaster* and *D. ananassae* . As shown in the dot plot and protein alignment ( Figure 1C- A; 1D-B), many of the dissimilarities between the *Roc1a-RC* protein sequence in *D. melanogaster* and *D. ananassae* are found in the latter portion of the first CDS, which is unique to only isoform *Roc1a-RC* , as isoforms *Roc1a-RA* and *Roc1a-RD* have spliced out this region. A *blastp* search of of Roc1a-PA in *D. ananassae* has 96.30% identity (E-value: 5e-76) with the protein-coding isoform Roc1a-PA in *D. melanogaster* , which is much higher relative to the 79.43% identity of the same search using Roc1a-PC. The significantly higher identity value for the other isoforms of *Roc1a* in *D. ananassae,* taken in the context of the protein alignment shown in figure 1D, provides further evidence that this is likely the correct ortholog, and that while the portion of the protein alignment unique to Roc1a-PC contains low sequence similarity, the remaining regions of *Roc1a* are very well-conserved.

## Methods

Detailed methods including algorithms, database versions, and citations for the complete annotation process can be found in Rele et al. (2023). Briefly, students use the GEP instance of the UCSC Genome Browser v.435 ( https://gander.wustl.edu ; Kent WJ et al., 2002; Navarro Gonzalez et al., 2021) to examine the genomic neighborhood of their reference IIS gene in the *D. melanogaster* genome assembly (Aug. 2014; BDGP Release 6 + ISO1 MT/dm6). Students then retrieve the protein sequence for the *D. melanogaster* reference gene for a given isoform and run it using *tblastn* against their target *Drosophila* species genome assembly on the NCBI BLAST server ( https://blast.ncbi.nlm.nih.gov/Blast.cgi ; Altschul et al., 1990) to identify potential orthologs. To validate the potential ortholog, students compare the local genomic neighborhood of their potential ortholog with the genomic neighborhood of their reference gene in *D. melanogaster* . This local synteny analysis includes at minimum the two upstream and downstream genes relative to their putative ortholog. They also explore other sets of genomic evidence using multiple alignment tracks in the Genome Browser, including BLAT alignments of RefSeq Genes, Spaln alignment of *D. melanogaster* proteins, multiple gene prediction tracks (e.g., GeMoMa, Geneid, Augustus), and modENCODE RNA-Seq from the target species. Detailed explanation of how these lines of genomic evidenced are leveraged by students in gene model development are described in Rele et al. (2023). Genomic structure information (e.g., CDSs, intron-exon number and boundaries, number of isoforms) for the *D. melanogaster* reference gene is retrieved through the Gene Record Finder ( https://gander.wustl.edu/~wilson/dmelgenerecord/index.html ; Rele et al *.,* 2023). Approximate splice sites within the target gene are determined using *tblastn* using the CDSs from the *D. melanogaste* r reference gene. Coordinates of CDSs are then refined by examining aligned modENCODE RNA-Seq data, and by applying paradigms of molecular biology such as identifying canonical splice site sequences and ensuring the maintenance of an open reading frame across hypothesized splice sites. Students then confirm the biological validity of their target gene model using the Gene Model Checker ( https://gander.wustl.edu/~wilson/dmelgenerecord/index.html ; Rele et al., 2023), which compares the structure and translated sequence from their hypothesized target gene model against the *D. melanogaster* reference gene model. At least two independent models for a gene are generated by students under mentorship of their faculty course instructors. Those models are then reconciled by a third independent researcher mentored by the project leaders to produce the final model. Note: comparison of 5' and 3' UTR sequence information is not included in this GEP CURE protocol.

## Data Availability

Description: A Zip file containing a GFF, FASTA, and PEP of the model. Resource Type: Model. DOI: https://doi.org/10.22002/je5dq-jg936
